# ID 266 - Terapia Gênica no Tratamento da Hemofilia A: uma revisão sistemática

**DOI:** 10.5327/2237-9622.2025.v34s1.266

**Published:** 2025-11-25

**Authors:** Ana Clara Silva Mendes, Ana Clara Silva Mendes, Luila Clicia Moura Henriques, Douglas Lamounier de Almeida, Vitoria Eduarda Alves de Jesus, Ricardo Mesquita Camelo, Juliana Álvares Teodoro

**Keywords:** hemofilia A, valoctocogene roxaparvoveque, giroctocogene fitelparoveque, dirloctocogene samoparvoveque, fator VIII, emicizumabe, profilaxia, revisão sistemática

## Abstract

**Introdução:**

A hemofilia A (HA) é um distúrbio hemorrágico hereditário caracterizado por deficiência do fator VIII (FVIII) da coagulação. A terapia das pessoas com HA (PcHA) envolve o tratamento de sangramentos (episódico) com FVIII exógeno ou prevenção dos sangramentos (profilaxia) com FVIII exógeno ou emicizumabe. A despeito da administração frequente desses produtos, as PcHA continuam apresentando sangramentos de escape e desenvolvendo artropatia secundária às hemartroses. A terapia gênica (TG) baseada em vetores virais carreadores de trangenes de F8 vem sendo estudada como tratamento definitivo das PcHA, uma vez que se pretende aumentar ou normalizar a expressão de FVIII por longo prazo ou toda a vida. Recentemente, alguns países aprovaram a TG com valoctocogene roxaparvoveque. Outras TGs em fases finais de estudos clínicos devem ser aprovadas nos próximos anos. Objetivo: avaliar a eficácia/efetividade e a segurança das TG em HA.

**Método:**

Uma revisão sistemática está sendo desenvolvida. Inicialmente, fez-se uma busca (16/6/2024) no envolvendo TG em HA, para identificar produtos que estejam sendo ou já tenham sido avaliados em estudos de fase 3 e estejam aprovados para uso clínico em algum país. Esses produtos constituíram unitermos na estratégia de busca (16/6/2024) realizada nas bases de dados bibliográficas PubMed, Embase, Cochrane e LILACS. A população definida foi homens adultos com HA grave ou moderada, sendo o grupo-controle tratado com profilaxia com FVIII ou emicizumabe. Incluíram-se ensaios clínicos randomizados e não randomizados, estudos observacionais, revisões sistemáticas e estudos que comparassem indiretamente com ajuste por correspondência (Ciac) TG com profilaxia com FVIII ou emicizumabe. Os desfechos avaliados envolverão taxas anualizadas de sangramento, consumo de FVIII, qualidade de vida e eventos adversos. As avaliações de risco de viés serão realizadas em seguida, e a qualidade da evidência será definida pelo método GRADE – do inglês, (PROSPERO: CRD42024562798).

**Resultados:**

A busca inicial sobre produtos de TG em HA obteve três resultados: valoctocogene roxaparvoveque, giroctocogene fitelparvoveque e dirloctocogene samoparvoveque. A busca nas bases de dados recuperou 331 publicações. Após a remoção das duplicatas e a seleção das publicações conforme os critérios de inclusão, 12 publicações entre 2017 e 2024 foram incluídas, envolvendo 4 estudos clínicos (n=11) e 1 Ciac. Os estudos clínicos foram realizados principalmente na Europa e na América do Norte. Os delineamentos compreenderam seis relatos de estudos clínicos não randomizados de fase 1/2 (valoctocogene roxaparvoveque = 4; giroctocogene fitelparvoveque = 1; e dirloctocogene samoparvoveque = 1) e seis relatos de estudos clínicos não randomizados de fase 3 (todos valoctocogene roxaparvoveque). Todos os estudos iniciais com valoctocogene roxaparvoveque tiveram análises, com descrição de dados de eficácia e segurança por até seis anos após a TG. O Ciac incluído comparou TG com valoctocogene roxaparvoveque com profilaxias com FVIII ou emicizumabe e foi publicado em 2023. As análises de desfechos, risco de viés e qualidade da evidência serão relatadas no congresso.

**Conclusão:**

Espera-se que nossas descobertas contribuam na síntese de evidências científicas dos tratamentos da HA e no progresso da avaliação de novas tecnologias em saúde no tratamento das PcHA.

